# Role of the gut–brain axis in energy and glucose metabolism

**DOI:** 10.1038/s12276-021-00677-w

**Published:** 2022-04-26

**Authors:** Hallie R. Wachsmuth, Savanna N. Weninger, Frank A. Duca

**Affiliations:** 1 Department of Physiology, University of Arizona, Tucson, AZ USA; 2 School of Animal and Comparative Biomedical Sciences, College of Agricultural and Life Sciences, University of Arizona, Tucson, AZ USA; 3 BIO5, University of Arizona, Tucson, AZ USA

**Keywords:** Obesity, Type 2 diabetes, Obesity

## Abstract

The gastrointestinal tract plays a role in the development and treatment of metabolic diseases. During a meal, the gut provides crucial information to the brain regarding incoming nutrients to allow proper maintenance of energy and glucose homeostasis. This gut–brain communication is regulated by various peptides or hormones that are secreted from the gut in response to nutrients; these signaling molecules can enter the circulation and act directly on the brain, or they can act indirectly via paracrine action on local vagal and spinal afferent neurons that innervate the gut. In addition, the enteric nervous system can act as a relay from the gut to the brain. The current review will outline the different gut–brain signaling mechanisms that contribute to metabolic homeostasis, highlighting the recent advances in understanding these complex hormonal and neural pathways. Furthermore, the impact of the gut microbiota on various components of the gut–brain axis that regulates energy and glucose homeostasis will be discussed. A better understanding of the gut–brain axis and its complex relationship with the gut microbiome is crucial for the development of successful pharmacological therapies to combat obesity and diabetes.

## Gut–brain communication: Role in energy and glucose metabolism

Signaling between the gut and the brain involves a complex mix of nutrients, peptides, and microbes that could be targeted as therapies for obesity and diabetes. Our bodies regulate food intake and energy expenditure via cells in the intestinal wall that sense certain nutrients, then release peptides which activate neurons. A review by Frank Duca and graduate students Hallie Wachsmuth and Savanna Weninger at the University of Arizona, Tucson, USA, reveals a more complicated picture, with many different interacting neural and hormonal signals, with increasing evidence for the vital role played by the microbes in the gastrointestinal tract. In particular, the gut microbiome impacts food intake, fat formation, endogenous glucose production, and insulin sensitivity. Novel metabolic therapies could target specific signaling pathways between the microbes, gut, and brain.

## Introduction

The prevalence of metabolic diseases, such as obesity and type 2 diabetes (T2D), is steadily rising worldwide, due in large part to a westernized lifestyle that favors increased energy intake of highly palatable foods coupled with decreased energy expenditure. In the United States, almost three-quarters of adults are considered overweight and 43% are obese, nearly tripling the prevalence of obesity observed 40 years ago^1^. Similarly, the worldwide prevalence of diabetes has more than doubled in the past 40 years, reaching 9.3% in 2019^2^. The health care cost associated with diabetes is equal to that of obesity, as are the rates of comorbidities and mortality^3^. Despite a dire need for the prevention and amelioration of these metabolic diseases, few successful therapeutic options exist. Although highly invasive, the most successful current treatment for obesity and diabetes is bariatric surgery^4^, highlighting the major impact of the gastrointestinal (GI) tract on energy and glucose homeostasis. In line with this, some of the more promising pharmacological therapies also involve modulation of the GI tract. For example, glucagon-like peptide-1 (GLP-1) agonists and dipeptidyl peptidase-4 inhibitors used for T2D treatment originate from gut-derived signaling mechanisms^5^. Even metformin, the most prescribed drug for T2D, acts at least partly via the gut to lower blood glucose via gut–brain–liver signaling mechanisms that lower hepatic glucose production or via alterations in gut microbiota and bile acid signaling^6–8^. Intertwined with the targeting of the GI tract for metabolic disease therapy, studies suggest that manipulations of the gut microbiome can directly impact host metabolic homeostasis^9^ and influence the success of other treatment options, such as gastric bypass surgery and metformin^6,8,10^. Taken together, these findings highlight the function of the GI tract in the maintenance of metabolic diseases and emphasize its role as a therapeutic target.

**Fig. 1:**
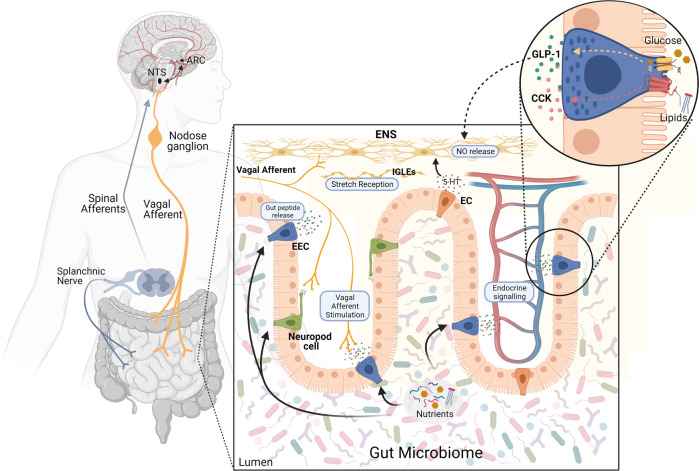
Major mediators of the gut–brain axis. Specialized intestinal epithelial cells, enteroendocrine cells (EECs), neuropod cells, and enterochromaffin cells (ECs), secrete gut peptides, including GLP-1, CCK, GIP, and PYY, on the basolateral side. These gut peptides are released in close proximity to vagal afferent neurons innervating the intestinal mucosa and activate these neurons. Vagal afferent neurons send signals to the nucleus tractus solitarius (NTS), which can send signals to higher-order brain regions, such as the arcuate nucleus (ARC). Vagal afferent neurons are also activated via the enteric nervous system, which can be activated by the release of gut-derived neurotransmitters, such as 5-HT, from ECs and intraganglionic laminar endings (IGLEs) sensing intestinal stretch. In addition, gut peptides are able to enter the circulation and carry signals directly to the NTS or activate splanchnic nerve endings to signal to the brain via spinal afferent neurons.

The gut–brain axis is a bidirectional hormonal and neural signaling pathway connecting the gut and the brain. As depicted in Fig. 1, several mechanisms link the gut to the brain in the regulation of metabolic homeostasis. Classically, signals originating from the gut in response to an influx of nutrients during a meal are relayed to the brain, informing the central nervous system (CNS) about meal size^11^ and composition^12,13^. The brain, mainly the hypothalamus, integrates these gut-derived signals, along with others, to coordinate the regulation of food intake, energy expenditure, and glucose homeostasis^14,15^. Enteroendocrine cells (EECs), specialized neuroendocrine cells of the intestinal epithelium, mediate this postprandial gut feedback, as they sense the luminal milieu and release gut peptides that influence metabolic homeostasis, such as GLP-1 and cholecystokinin (CCK)^16^. Nutrient-induced gut peptides can act in a paracrine fashion by activating vagal neurons that innervate tissue near the intestinal epithelium and signal to the brain or in an endocrine fashion targeting the brain and other peripheral organs involved in maintaining metabolic homeostasis^17^. Despite the established significance of postprandial gut–brain signaling in energy and glucose homeostasis, recent work highlights the physiological and neural complexity of this negative feedback and questions this gut peptide-mediated vagal signaling. In the current review, we will provide an overview of how the gut–brain axis contributes to metabolic homeostasis, highlighting some of the novel ideas as to how this gut–brain communication is mediated. Furthermore, this traditional, nutrient-induced, neural, and hormonal gut–brain signaling pathway fails to incorporate the role of the gut microbiome in mediating gut-derived feedback mechanisms. Given the high numbers of microbial cells and genes^18^, it is now evident that the gut microbiome can impact glucose and energy homeostasis via numerous mechanisms, including both impacting host gut–brain signaling and initiating direct communication to the brain via microbe-derived metabolites^19^. This review will highlight the current ideas as to how the gut microbiome incorporates into host gut–brain signaling and the impact of gut bacteria on host energy and glucose homeostasis via CNS signaling pathways.

## Neuronal gut–brain connection

Gut–brain signaling is a bidirectional avenue of communication. Signals from the brain communicate to the gut mainly through the autonomic nervous system and the hypothalamic–pituitary axis to regulate many physiological processes (reviewed elsewhere^20^). Neural signaling from the gut to the brain, however, is mediated by vagal and spinal afferent neurons^21,22^. Spinal afferents innervate the intestine, but their role in energy and glucose homeostasis appears minimal^23,24^, although a more recent study revealed a role for spinal afferents in mediating intestinal sensing of glucose^22^. In addition, the enteric nervous system (ENS), which is a recognized “second brain” that can regulate many GI functions independent of CNS action, can also act as an intermediate in relaying gut-derived information to the CNS^25^. The following section will focus on the role of the vagus nerve and ENS in regulating metabolic homeostasis.

### Vagus nerve

The vagus nerve is aptly nicknamed the “wanderer nerve” due to long extensions originating from the brainstem and innervating many of the visceral organs. Within the gut, vagal afferent endings are distributed in different layers, with many innervating the lamina propria of the GI tract near EECs^26,27^. Vagal afferent neurons (VANs) innervate the entire GI tract, although recent studies have discovered a distinct heterogeneity of VANs, with different subgroups more densely populating different anatomical locations of the GI tract^27–32^. The gut stimulates VANs in two main ways: via chemoreception or mechanostimulation^32^. Vagal mucosal endings are better situated to sense chemical stimuli, while sensing of GI stretching and distention is accomplished by intraganglionic laminar endings (IGLEs) that act as mechanoreceptors^26,29,30^. Important for the regulation of energy and glucose homeostasis, a high number of VANs contain receptors for gut peptides released by EECs, and the physiological function of these VANs has been recently elucidated via new advances in the ability to characterize and target specific neural populations. Indeed, recent studies have found that over 10 distinct cell types likely reside in the nodose ganglion according to the expression profile of various proteins and receptors, but the results are sometimes contradictory^28,31,32^. As such, one paper detailed over 15 different subtypes of vagal neurons that innervate the viscera^31^, while another study demonstrated 12 unique clusters specifically originating from the GI tract^28^. This newly recognized heterogeneity in vagal neuron protein expression likely impacts the functions of specific neurons, as detailed below, and requires further investigation. However, it is recognized that vagal terminals activated within the GI tract send signals up to the brain; the signals are processed within cell bodies contained in the nodose ganglia and ultimately reach the nucleus tractus solitarius (NTS) and the area postrema within the hindbrain. From the hindbrain, the information can be relayed to various local brain regions, including the vagal dorsal motor nucleus and parabrachial nucleus, and eventually to higher-order brain centers, such as the hypothalamus. In particular, signals from the gut can activate specialized neurons of the arcuate nucleus (ARC) that regulate energy and glucose homeostasis, including agouti-related peptide/neuropeptide Y orexigenic neurons and pro-opiomelanocortin/cocaine and amphetamine-regulated transcript anorexigenic neurons^22,33–35^. Importantly, the left and right branches of the vagal nerve are not symmetric and are unique in where they innervate within the GI tract and where they terminate in the hindbrain^28^. For example, the common hepatic branch of the vagus diverges from the left subdiaphragmatic vagus trunk to innervate the intestines and liver, while both the left and right trunks contain celiac branches that innervate the distal intestine^36^. Accordingly, recent research demonstrates distinct differences in the function of neurons within the left vs. right nodose ganglia in the regulation of energy homeostasis, as the right nodose ganglia of mice mediate the rewarding response to nutrients, while the left nodose ganglia control satiation independent of reward^37^.

The vagus nerve relays information regarding a meal to the brain to regulate both energy and glucose homeostasis^13^. Indeed, VANs respond to nutrients sensed in the gut, as vagal afferent activity is increased during infusion of either carbohydrates, amino acids, or fatty acids into the small intestine^12,38–40^. Likewise, c-fos, a marker of neuronal activity, is increased in the NTS, where vagal afferents terminate, and in the nodose ganglia after different nutrient loads^41^. It is possible that this is due to the nutrient-induced release of gut peptides (see the section below); however, using genetic approaches to target specific neurons, a recent study challenges that idea^32^. Williams et al. demonstrated that contrary to expectations, subsets of VANs containing GLP-1 receptor (GLP-1R) detected stomach and intestinal stretch and, interestingly, had no impact on nutrient sensing, which was instead regulated by G protein-coupled receptor 65 (GPR65)-positive neurons^32^. Conversely, Bai et al. demonstrated no effect of optogenetic or chemogenetic activation of GPR65-positive cells on food intake and only a mild effect via GLP-1R-positive cells^28^. Instead, only activation of *Oxtr*-containing neurons, exclusively intestinal IGLEs that detect stretch, potently suppressed food intake, while various subtypes of mucosal ending neurons, which are classically thought to sense gut peptides, had no effect^28^. However, gut peptide signaling could influence IGLE neuronal signaling, as these neurons express gut peptide receptors. Despite this, the salient role of overall vagal signaling in energy homeostasis has been established, as surgical or chemical ablation of vagal afferent signaling decreases acute sensitivity to intestinal nutrients, leading to increased food intake and reduced NTS neural activation^23,24,42–44^. In addition, chronic stimulation of VANs reduces food intake and weight gain in rats^45–47^ and is associated with weight loss in humans^48^, highlighting the long-term physiological relevance of vagal signaling.

In addition to food intake, vagal afferents also mediate the ability of intestinal nutrients to regulate hepatic glucose production. Infusion of lipids and carbohydrates into the small intestine initiates a gut–brain–liver axis that is dependent on vagal signaling to lower hepatic glucose production and improve glucose tolerance^49^. Interestingly, this effect was mediated by the release of CCK and GLP-1 and subsequent activation of their receptors, possibly on VANs (see “Hormonal gut-brain signaling” section). However, as mentioned previously for vagal afferent regulation of food intake, recent work has failed to demonstrate an effect of food intake via direct gut peptide signaling on VAN-localized receptors. Given the increased ability to identify and target specific neural populations, future studies will need to more comprehensively address the effects of IGLEs and mucosal ending VANs on both energy and glucose homeostasis. Indeed, it is puzzling why only IGLE neurons detecting intestinal distention induce satiation, while mucosal ending VANs that contain gut peptide receptors and are ideally situated in close proximity to EECs would not be involved in metabolic homeostasis.

Although it is possible that vagal afferents are activated directly within the GI tract, several studies have highlighted the role of vagal nutrient sensing in the portal vein^50,51^. For example, glucose is absorbed by the intestine and enters the portal vein, where glucose sensing increases hepatic vagal signaling to the NTS^52^ and subsequently to the hypothalamus^53^ to decrease food intake^54,55^ and regulate glycemia^56,57^. Furthermore, the GI tract generates glucose via gluconeogenesis, and it has been shown that both proteins and fiber increase gluconeogenic enzymes in intestinal epithelial cells. This increase in intestinal gluconeogenesis leads to portal glucose sensing to regulate energy homeostasis and improve insulin sensitivity^58^. Although not fully understood, nutrients likely first activate CNS mechanisms to induce intestinal gluconeogenesis via release of vasoactive intestinal peptide by the ENS. These peptides then increase intestinal glucose-6-phosphotase, ultimately leading to activation of a portal glucose sensor that signals to the brain^59^. However, this portal glucose sensor may still require gut input, as these effects appear to be mediated by vagal GLP-1R activation^60–62^.

Interestingly, impaired vagal signaling may play a role in obesity, as decreased vagal activation to both mechanical stimuli and satiety-inducing signals is characteristic of a high-fat diet (HFD)^63–66^. One study suggests that the decrease in vagal signaling seen with HFD feeding is due to upregulation of K+ channels in the nodose ganglia, increasing hyperpolarization of VANs^67^, while other studies have implicated changes in the gut microbiota as a contributor to vagal afferent desensitization during HFD feeding (see the “Gut microbiota–brain connection” section). For a more comprehensive review on impaired gut signaling during obesity, see ref. ^68^.

### Enteric nervous system

The ENS is an independently functioning neural system innervating the entire GI tract and containing two bundles of ganglia: one underneath the epithelial layer of the gut (submucosal plexus) and the other between the smooth muscle layers of the gut (myenteric plexus)^25^. One of the main roles of the ENS is mechanoreception and stimulation of the smooth muscle surrounding the gut^69,70^, providing autonomous control of GI transit and motility. In regard to mediating energy or glucose homeostasis, neurons of the myenteric plexus of the ENS are activated via duodenal glucose infusion^71^ and by hormones released in response to nutrient sensing in the gut, such as GLP-1 from EECs and serotonin (5-HT) from enterochromaffin cells (ECs)^71–74^. Indeed, enteric neurons express GLP-1Rs, and primary cultures of myenteric neurons have increased neuronal activation following GLP-1 treatment^73^. Given that vagal afferents innervate the ENS, it is plausible that the ENS can relay local intestinal signaling mechanisms to the CNS. For example, enteric neurons express glutamate transporters and are in close proximity to VANs expressing glutamate-sensitive receptors^75^. Although VANs are activated by glutamate^76–78^, the link between glutamate release by the ENS and the stimulation of VANs has yet to be proven^76–78^. Nonetheless, GLP-1-induced gastric emptying and insulin secretion depend upon ENS GLP-1R sensitivity and subsequent relay to the hindbrain via enteric nitric oxide (NO) production^72^. Interestingly, GLP-1 resistance during HFD feeding reduces enteric activation and NO production, which is due to changes in the gut microbiota (see the “Gut microbiota–brain connection” section)^72^. Along these lines, increased duodenal contractility by the ENS is characteristic of T2D and likely contributes to metabolic dysfunction. Treatment with galanin or apelin, which are both peptides released in the intestine, results in decreased duodenal contraction via the ENS, which subsequently signals to the hypothalamus, leading to increased hypothalamic NO release and whole-body glucose utilization^79,80^. Taken together, evidence suggests that the ENS may mediate gut–brain signaling that regulates metabolic homeostasis.

## Hormonal gut–brain signaling

The GI tract is a highly specialized organ system that is sophisticated beyond its functions of digestion and absorption. Indeed, while the gut is responsible for providing the host with required nutrients from the diet, it is also home to hormonal, neural, and microbial systems that can have significant impacts on the host^19^. Despite the small percentage (~1%) of intestinal epithelial cells classified as EECs, they constitute the largest endocrine organ in the body and are present throughout the entire GI tract, increasing in density distally^81^. Most EECs are considered open-type cells, resembling a bottleneck shape, with an apical membrane in direct contact with the intestinal lumen, as well as a basolateral membrane in close proximity to blood vessels and neurons that innervate the intestinal mucosa and come in close contact with the epithelial layer^82^. EECs contain peptides (also sometimes termed hormones) in secretory granules, and upon chemical, mechanical, or neural stimulation, an influx of intracellular calcium stimulates the secretion of gut peptides through the basolateral membrane and into the extracellular space^17^. EECs are classically characterized based on the gut hormones they secrete, such as L-cells (GLP-1), I-cells (CCK), and K-cells (GIP)^83^. However, recent advances in EEC-specific transgenic mouse models and single cell RNA sequencing (RNA-seq) studies suggest that EECs exhibit a complex phenotype with multiple hormones expressed in specific EECs and that the similarity of subtypes depends more on EEC location^81^. For example, one study examined cells expressing preproglucagon, a precursor of GLP-1, in the upper small intestine of mice and found three separate clusters of cells with overlapping but distinct hormonal profiles^84^. Interestingly, the clusters differed not only in hormonal expression but also in the expression of receptors and ion channels, highlighting the potential specificity of targeting these unique EEC clusters^84^. More recently, intestinal organoid models and real-time EEC reporter mice have demonstrated the complexity and heterogeneity of EEC differentiation and gene expression^85,86^. Similar to previous reports, many EECs were multihormonal^81,87^, and many subtypes within EECs may not be distinct lineages but rather are separate stages in EEC maturation^86^. For example, as cells with high levels of *Gcg* expression decreased, a concomitant rise in cells expressing *Cck* was observed at the same timepoint, indicating a hormonal shift in the same EECs^85,86^. This unique maturation of EECs is unlike the more traditional maturation of enterocytes, which move from the crypts up the villi^88^. Importantly for the translational impact of potential EEC-targeted therapies, comparisons between murine and human EECs via RNA-seq have established that receptors expressed in murine models are largely equivalent to those seen in humans^89^. However, despite this similarity, whether human EECs follow a similar shift in uniqueness during various maturation stages remains to be assessed. Nonetheless, these studies taken together challenge the classical idea of EEC cell types and indicate that EECs are a rare and complex class of cells that secrete peptides based largely on their location and maturation within the gut^81,84,89–91^.

Given their open-type nature, EECs are uniquely suited to act as initial messengers regarding incoming nutrients. This allows the host to prepare for an influx of nutrients to maintain energy and glucose homeostasis, and interestingly, obesity and HFD feeding are associated with impaired postprandial gut–brain signaling, highlighting the pathophysiological impact of these signaling pathways (for extensive review, see, ref. ^92^). EECs highly express chemosensory machinery compared to non-EEC intestinal epithelial cells, including nutrient-specific G protein-coupled receptors (GPCRs) and nutrient transporters, enabling them to sense and respond to the intestinal milieu^17^. The specific mechanisms regulating nutrient-induced gut peptide release from EECs are still under investigation but appear more complicated than originally hypothesized^17^. Although EECs might sense nutrients via activation of specific GPCRs on the apical side, where they are in direct contact with the luminal environment, elegantly performed ex vivo and in vitro studies suggest that many nutrients, including fatty acids, may be detected on the basolateral surface^17,93,94^.

There are over 20 identified gut peptides within the GI tract, many of which play a key role in energy and glucose homeostasis, with the following sections focusing on the incretin hormones GLP-1 and glucose-dependent insulinotropic polypeptide (GIP), CCK, and several others.

In addition to the more traditionally characterized EECs that secrete gut peptides, recent studies have discovered the presence of specialized EECs with long cytoplasmic processes on the basal side that directly signal primary afferent neurons innervating the gut^95^. These “neuropods” synapse and thus establish direct connections with vagal afferents, enteric glia, and efferent fibers, releasing neurotransmitters such as glutamate. Many EECs have neuropods and are heterogeneous depending on the GI tract region. First discovered in distal intestinal peptide YY (PYY)-containing EECs^95^, neuropod cells in the intestine can sense sugars and transduce a signal to the brainstem within milliseconds via a single synapse^96^. This rapid signal, mediated by neuropod cell release of glutamate onto VANs, provides nearly instantaneous spatial and temporal information on a meal, which can then be followed by more traditional gut peptide–vagal afferent signaling pathways outlined below^95,96^.

### Incretin hormones: GLP-1 and GIP

**Fig. 2:**
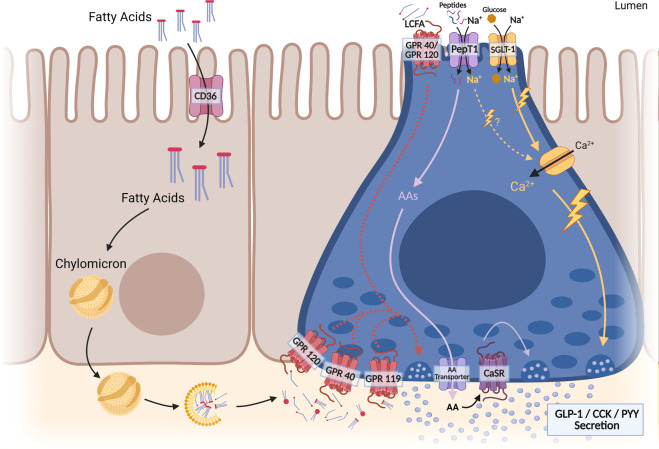
Nutrient sensing by enteroendocrine cells. The different macronutrients act through alternate pathways to elicit gut peptide release. Fatty acids can signal through multiple receptors on both the apical and basolateral membranes. Signaling at the basolateral membrane requires the uptake and packaging of fats into chylomicrons in enterocytes followed by the release and breakdown of these chylomicrons at the basolateral surface. Fatty acids bind their receptors on enteroendocrine cells, which activate a downstream signaling cascade leading to the fusion of gut peptide–containing vesicles and the release of their contents at the basolateral membrane. Glucose sensing occurs at the apical membrane of an EEC and requires uptake into the cell, along with Na^+^, via SGLT-1. Na^+^ entry into the EEC causes depolarization and subsequent activation of Ca^2+^ channels, resulting in vesicle fusion and gut peptide release. Amino acid signaling in the enteroendocrine cell involves the uptake of peptides and Na^+^ via PepT1 at the apical membrane. This Na^+^ may depolarize the cells, but research is still needed to determine its exact mechanism of action. Once a peptide enters the cell, it is broken down into amino acids, which are transported out of the cell through the basolateral membrane, where they can activate CaSR, leading to Ca^2+^ release and vesicle fusion. CaSR may also be present at the apical membrane, but research is still needed to elucidate the exact mechanism of protein-induced gut peptide release.

The incretin hormones GLP-1 and GIP are secreted by EECs in response to ingested nutrients and stimulate insulin secretion^97,98^. Although GLP-1 expression is highest in the large intestine, a large fraction of GLP-1-secreting EECs populate the small intestine^16^. Direct nutrient stimulation may induce the initial release of GLP-1 from small intestinal EECs, while large intestinal EECs likely release GLP-1 via a neural hormonal reflex or from microbe-derived metabolites, although this remains to be explored^93,99^ (see the “Gut microbiota–brain connection” section). As such, GLP-1 is present in circulation at low levels in fasting humans and begins to increase in the first few minutes after nutrient ingestion, peaking ~60–90 min postprandially at ~15 pmol/L of active GLP-1^97,100^. The release of GLP-1 from nutrient stimulation is ultimately dependent upon calcium influx, but macronutrients likely differ in their signaling mechanisms (Fig. 2)^101,102^. For example, glucose-stimulated GLP-1 release requires sodium/glucose cotransporter-1 induction of inward currents, initiating depolarization of EECs and calcium influx to release GLP-1^103–105^. Lipids, on the other hand, likely activate long-chain fatty acid (LCFA) receptors (GPR40 and GPR120) on the basolateral side, indicating that absorption and chylomicron formation are prerequisites^106–109^. The LCFA receptors GPR120 and GPR40 are colocalized in GLP-1-containing cells. However, GPR40 but not GPR120 knockout mice exhibit impaired GLP-1 secretion from dietary fat, and only GPR40 agonists induce GLP-1 release in vitro and in vivo^106,110–112^. In addition to LCFAs, 2-monoacylglycerol, the other component of triglycerides, can also induce GLP-1 release via G protein-coupled receptor 119^113–115^. Amino acid stimulation of GLP-1 is not fully understood but likely involves calcium-sensing receptor (CaSR) and peptide transporter 1 (PepT1)^116,117^.

Oral glucose administration results in up to three times more insulin being released than direct β-cell glucose sensing, highlighting the glucoregulatory impact of glucose-induced GLP-1 and GIP release from EECs^118,119^. Although β-cells express GLP-1R, and it is likely that GLP-1 induces insulin secretion directly in the pancreas^120^, GLP-1 has an extremely short half-life of ~1–2 min; thus, only ~12% of gut-derived GLP-1 enters the systemic circulation intact^121–123^. This highlights the possibility that GLP-1 could activate VANs that innervate either the gut or the hepatoportal region to signal to the brain and back down to the pancreas via vagovagal reflexes to induce insulin secretion^124^. Indeed, the common hepatic branch, as well as celiac and gastric branches, have all been implicated in contributing to the glucoregulatory effects of gut GLP-1^62,125,126^, although selective knockdown of GLP-1R in nodose ganglia does not impair oral glucose tolerance or glucose-stimulated insulin secretion, similar to genetic knockout of GLP-1R in vagal neurons^127,128^. As such, selective restoration of islet and pancreatic duct GLP-1R was sufficient to improve impaired oral glucose tolerance in global GLP-1R knockout mice, although there was no difference in glucose-stimulated insulin release^129^. Thus, the extent to which the gut–brain axis contributes to the incretin effect of GLP-1 remains to be determined. Despite this, GLP-1 can impact glucose homeostasis via a gut–brain neuronal axis, independent of changes in insulin secretion.

As described earlier, infusion of both lipids and glucose into the small intestine leads to an improvement in glucose tolerance via a reduction in hepatic glucose production. Intestinal glucose infusion increases portal levels of GLP-1, and coinfusion of exendin-9, a GLP-1R antagonist, abolishes the ability of both intestinal fatty acids and glucose infusion to lower hepatic glucose production^130,131^. In addition, intestinal lipid infusion lowers hepatic glucose production via a vagal afferent and subsequent hepatic vagal efferent pathway^49^. Thus, endogenous GLP-1 likely acts on GLP-1R expressed in vagal afferents^128^. Indeed, nodose GLP-1R lentivirus-mediated knockdown increases postprandial glycemia^128^, and *Glp1r*^ΔPhox2b−/−^ mice, which exhibit selective knockout of GLP-1R in the nodose ganglia, midbrain, and hindbrain, exhibit increased fasting levels of glucose, both highlighting a role for GLP-1 vagal afferent signaling in mediating glucose homeostasis^127^. However, it is possible that GLP-1 acts on other local cell types that can subsequently activate VANs, such as the enteric neurons described earlier^71–74^. Given the short half-life of endogenous GLP-1, local vagal signaling likely mediates the effect of intestinal GLP-1 release on food intake as well^123^.

In addition to a glucoregulatory effect, exogenous GLP-1 and GLP-1R agonists reduce food intake^132–134^. However, caution must be taken when examining exogenous gut peptide studies, as many times the dose is supraphysiological, and while there may be therapeutic relevance, it may not be an accurate representation of endogenous gut peptide action. Thus, throughout the manuscript (as with GLP-1 glucoregulatory function above), we aim to highlight studies that directly examine endogenous gut peptide action via receptor knockdown or antagonism. However, when possible, we identified the dosage of exogenous gut peptide administration and whether it is a plausible representation of endogenous levels. For example, capsaicin treatment, which destroys some vagal afferents, abolishes the suppressive effect of low levels of GLP-1 administration (6 and 120 pmol/kg)^135^. Furthermore, vagotomy abolishes the effect of GLP-1 on food intake, while more selective subdiaphragmatic vagal deafferentation attenuates the inhibitory effect of higher doses of intraperitoneal GLP-1 (100 nmol/kg)^136^. Long-term administration of exendin-9 abolishes the feeding-suppressive effect of small intestinal lipid infusion, indicating a role of nutrient-induced endogenous GLP-1 in food intake^130,137^. As such, selective vagal afferent GLP-1R knockdown in rats results in increased meal size and gastric emptying. However, there is no overall impairment in long-term energy balance, similar to *Glp1r*^ΔPhox2b−/−^ mice, which have similar body weight, adiposity, and long-term food intake to controls on either chow or HFD^127^. Thus, although it is likely that endogenous GLP-1 signals via a vagal gut–brain axis to lower meal size, the impact of this signaling pathway on overall energy homeostasis may be limited.

GIP acts on the pancreas, stimulating the release of insulin and glucagon to regulate blood glucose^138,139^. However, the effect of GIP on energy homeostasis is contradictory. In addition to GIP’s role in promoting lipid storage in adipocytes^140^, the GIP receptor (GIPR) is found in neurons of the ARC, dorsomedial nucleus (DMH), and paraventricular nucleus of the hypothalamus^141^. Thus, GIP could act in an endocrine fashion to impact food intake. Some studies suggest that central GIP signaling induces body weight gain and adiposity, possibly via induction of neural leptin resistance^142^. In humans, GIP secretion is positively correlated with obesity^100,143,144^. Furthermore, increases in GIP are seen with HFD consumption in humans and rodents; in humans, this occurs even before changes in body weight^145,146^. In line with this, some research suggests that antagonism of GIP receptors is beneficial for decreased weight gain^147,148^ and that CNS GIPR knockout mice are protected from diet-induced obesity^149^. On the other hand, more recent work suggests that GIP analogs lower body weight via GIPR agonism^150,151^. Activation of hypothalamic GIPR cells reduces food intake, while the suppressive effects of central and peripheral administration of acyl-GIP on food intake are abolished in CNS GIPR knockout mice^149^. Thus, while direct gut–brain signaling of GIP contributes to energy homeostasis, it is still unclear whether endogenous GIP is beneficial, and this unresolved discrepancy remains puzzling and requires further investigation^152^.

### CCK

CCK is released from EECs of the upper small intestine in response to fats and proteins. GPR40 mediates fatty acid sensing for CCK release, whereas protein sensing is mediated by CaSR in the proximal small intestine^153–155^. Traditionally, EECs are thought to release CCK through GPR40 activation, which induces cellular depolarization through intracellular calcium store release by inositol triphosphate–dependent mechanisms^17^. However, similar to the release of GLP-1, there may be a role for cluster of differentiation 36-mediated lipid absorption, chylomicron formation, and basolateral EEC activation in CCK release (Fig. 2)^156^. In healthy humans, CCK released from EECs enters the circulation and peaks in concentration from 6 to 15 pmol/L at ~90–120 min postprandial depending on fat content and meal form^157,158^.

CCK is a peptide differentially cleaved from pro-CCK by prohormone convertases into various forms depending on cleavage length, which have been isolated in the GI tracts of various species^159^, each thought to exert slightly different effects on energy homeostasis^160^. For example, while CCK-8, the most abundantly utilized form, reduced meal size when given exogenously (at various nmol/kg doses), CCK-58, the major endocrine form in rats and humans, reduced meal size and increased the interval time between meals^161^. Furthermore, the effects of the various forms of CCK, namely, CCK-8, 33, and 58, differ depending on their site of action in the gastrointestinal tract^162–164^. However, in studies, the most frequently administered exogenous form is CCK-8^165^, despite it not being the major endocrine form. Unfortunately, it is extremely difficult to measure the different forms, and most studies cannot differentiate the concentration of specific CCK forms^166–169^. Thus, moving forward, future studies should attempt to be more specific in the cleavage form when utilizing CCK in their hypothesis and experiments, as the effects of the different forms differ greatly. Nonetheless, endogenous and exogenous CCK (mostly CCK-8) acts on VANs to decrease meal size^170,171^. VANs innervating the GI tract contain CCK-1 receptor (CCK-1R) mRNA and respond to CCK-8 ex vivo^12,14,172–174^. Low levels of exogenous CCK-8 administration (4 ng/kg/min IV) have also been shown to decrease food intake in both lean and obese humans^175^, and intraperitoneal injection of CCK-8 decreases food intake in rats in a dose-dependent manner^176^. Slightly higher doses of CCK-8 administered via intraperitoneal injection (~1–10 µg/kg) activate vagal afferents and increase neuronal activation in the NTS where VANs terminate, while vagotomy and capsaicin treatment abolish the suppressive effects of CCK-8 administration^177,178^. Furthermore, knockdown of CCK-1R in vagal afferents via nodose ganglia lentiviral injection abolishes the ability of exogenous CCK-8 (2.5 µg/kg) to lower food intake^179^. Given that exogenous CCK, at potentially similar levels to postprandial endogenous CCK, activates vagal signaling pathways to lower food intake, it is likely that endogenous CCK mediates the suppressive effects of nutrients.

Lipids potently increase CCK release^180,181^, and administration of CCK-1R antagonists blocks the satiating effects of intestinal lipid infusion in both rodents and humans^169,182^. In support of a role for vagus-mediated CCK signaling, peripheral, but not central, antagonism of CCK-1R blocks the inhibitory effects of intestinal oleate and attenuates hindbrain activation^183^. The ability of lipids to induce CCK release and activate VANs to lower food intake may be due to chylomicron formation and subsequent activation of sensory machinery on the basolateral side of EECs, as mentioned previously. For example, coinfusion of lipids with a lipase inhibitor blocks the ability of lipids to increase CCK^166–169,184,185^ and ultimately suppress food intake^168,169^. Furthermore, treatment with Pluronic L-81, a surfactant that inhibits chylomicron formation, significantly attenuates the anorectic effects of intraduodenal lipids and attenuates both CCK release and celiac and cervical vagal afferent activation following lipid infusion^40,109,156,186^. Similarly, in humans, infusion of fatty acids with a chain length of less than C10, which do not assemble in chylomicrons but directly diffuse out of enterocytes, fails to reduce energy intake and hunger and does not increase circulating CCK levels^169^.

In addition to lipids, intestinal protein infusion significantly reduces food intake^187^ and increases CCK release. Several studies suggest that similar to lipids, reductions in food intake via intestinal protein in rodents are due to CCK release and subsequent activation of VANs, as (1) intestinal protein infusion increases CCK in rodents and humans^188,189^; (2) CCK-1R knockout rats exhibit a blunted response to peptone^190^; (3) peripheral chemical CCK antagonism abolishes the suppressive effects of peptone^191^; (4) intestinal peptones induce vagal afferent firing^192^; (5) capsaicin, a neurotoxin that selectively destroys small unmyelinated primary sensory neurons, abolishes the suppressive effect of amino acid L-phenylalanine^43^; and (6) vagotomy reduces the suppressive effect of L-phenylalanine^193^. Taken together, the evidence indicates that CCK vagal signaling likely mediates the inhibitory effects of food intake via intestinal nutrients.

Although the glucoregulatory effects of CCK are less studied than the suppression of food intake, CCK-8 can activate a neuronal signaling axis to lower hepatic glucose production^194^. Small intestinal lipid infusion lowers hepatic glucose production via a vagal gut–brain–liver axis that is dependent upon CCK-1R signaling, although recent evidence suggests that GLP-1R signaling also contributes to the effect^130,194^. Nonetheless, direct administration of CCK-8 also lowers hepatic glucose production during a glucose clamp^195^. Furthermore, in a more physiological setting, CCK-1R antagonism during a fasting-refeeding paradigm results in hyperglycemia, highlighting the glucoregulatory relevance of CCK-8 signaling^194^. In addition, CCK signaling may contribute to the pathophysiology of diabetes, as short-term HFD consumption abolished the ability of intralipid or low levels of exogenous CCK-8 (35 pmol/kg/min) to lower glucose production in rats during a glucose clamp^49,194^. However, upper small intestinal activation of protein kinase A (PKA) bypasses CCK resistance in regulating glucose homeostasis, although the role of PKA in feeding regulation remains unclear^196^. It is likely that CCK-1R signaling activates vagal afferents at least partly via PKA, as (1) CCK-1R activation increases cyclic adenosine monophosphate^197^, (2) PKA is necessary for CCK to activate central afferent terminals^172^, and (3) duodenal PKA activation leads to vagal afferent firing^196^. Overall, there is strong evidence suggesting that CCK can impact energy and glucose homeostasis by activating a gut–brain vagal pathway.

### Other gut-derived peptides

The previously mentioned gut peptides (GLP-1, GIP, and CCK) have been extensively studied for their role in metabolic homeostasis. However, the GI tract secretes numerous other peptides in response to ingested nutrients that may also play a role in energy and glucose homeostasis. PYY is a peptide released from colonic EECs in response to nutrients, with PYY3-36 being the most prevalent form of this gut peptide in the circulation^198,199^. The predominant role of PYY is thought to be the maintenance of energy intake, as it is a salient signal of satiety, although some studies have suggested a role for PYY in glucose homeostasis and insulin sensitivity^200–202^. The mechanism mediating the ability of PYY to regulate food intake is contentious but likely involves alterations in gastric emptying, vagal neuronal signaling to the hypothalamus^200,203,204^, and direct endocrine activation of the ARC^200^.

Oxyntomodulin (OXM) is a gut peptide produced by the colonic EECs that acts via GLP-1Rs and glucagon receptors^205,206^ to reduce body weight^207,208^ and improve glucose metabolism^209,210^. OXM exerts its homeostatic effects on energy balance by increasing energy expenditure via the glucagon receptor and decreasing energy intake, likely through hypothalamic and area postrema activation by GLP-1R signaling^207,211^. The mechanism for action for regulating glucose metabolism of OXM remains elusive but might involve a similar pathway to GLP-1 since it activates the same receptor, although OXM is still able to improve glucose metabolism in mice lacking GLP-1R^211^.

Ghrelin and nesfatin are gut peptides secreted primarily from gastric endocrine cells^212–214^ that induce opposing effects on energy homeostasis and metabolism. Ghrelin, commonly called the “hunger hormone,” is a potent stimulus for food intake in both humans^215^ and animals^216,217^; consistent with this, circulating levels are elevated during fasted and calorie-restricted states and decreased upon feeding^216,218^. Both central and peripheral administration of ghrelin increases food intake, body weight, and adiposity in rodents^216,217,219^. In addition, peripheral ghrelin administration at a dose almost double physiological levels (5 pmol/kg/min) increased acute food intake by nearly 30% in humans^215^. Ghrelin likely exerts these effects through both suppression of VAN firing and endocrine signaling in the ARC. In support of ghrelin’s proposed actions through VANs, ghrelin receptors are expressed on VANs, and subdiaphragmatic or gastric vagotomy abolishes increased food intake following peripherally administered ghrelin (1.5 nmol)^220^. In addition to regulating hunger, one study has strongly implicated peripheral ghrelin as a regulator of fasting blood glucose levels; global ghrelin receptor knockout mice exhibit low fasting blood glucose levels, which are increased when ghrelin receptor expression is selectively restored in nodose ganglia^221^. However, ghrelin receptors are expressed in the ARC^222^, and ghrelin can cross the blood–brain barrier^223^, although ghrelin transport from the blood to the brain might be too low to exert effects^223^. Therefore, it is hypothesized that ghrelin synthesized within the hypothalamus, as opposed to gut-derived ghrelin, is the mediator of central orexigenic effects of ghrelin, although further research is needed^224^.

In contrast to ghrelin, gastric endocrine cells also release an anorexigenic peptide, nesfatin^214,225^. Nesfatin is cleaved into three different forms: nesfatin-1, nesfatin-2, and nesfatin-3^226^. Of these, only nesfatin-1 has been established as a modulator of food intake^226^ and glucose homeostasis in rodents^227^. One study demonstrated that intraperitoneal injection of nesfatin-1 decreased food intake in a dose-dependent manner in mice and that c-Fos expression was significantly increased in the NTS, suggesting the role of VANs in this effect^228^. However, nesfatin-1 crosses the blood–brain barrier in a non-saturable fashion^229,230^. As such, centrally, but not peripherally, administered nesfatin-1 reproducibly causes reductions in food intake in rodents^231,232^. Furthermore, 2 weeks of subcutaneous infusion of nesfatin-1 improves glucose tolerance and insulin sensitivity in chow-fed and HFD-fed mice^227^, likely due to the direct action of nesfatin-1 on beta cells and not gut–brain signaling, similar to other peptides, such as GLP-1 and CCK^227,233,234^. However, circulating levels of nesfatin are generally not known; thus, despite the therapeutic potential, the effect and mechanism of endogenous nesfatin is still poorly understood.

Insulin-like peptide 5 (Insl5) is an orexigenic peptide released by EECs of the colon. As such, Insl5 plasma concentrations are increased in calorie-restricted states and suppressed during refeeding^235^. Likewise, Insl5 is decreased in energy-rich states such as high-fat feeding^236^. Due to the close proximity of EECs that produce Insl5 and enteric neurons expressing its receptor (relaxin/insulin-like family peptide receptor 4)^235^, Insl5 likely increases food intake via peripheral neuron signaling to the CNS. In support of this, peripheral, but not central, administration of high levels of Insl5 elicits an increase in food intake^235^. Consistent with this, Insl5 increases food intake in a dose-dependent manner in nonfasted control mice, but these effects are not preserved in relaxin/insulin-like family peptide receptor 4 knockout mice, receptors expressed on enteric and vagal afferent neurons but not in the hypothalamus^235^. However, peripheral Insl5-induced activation of brain areas associated with regulating food intake has yet to be established. Relatively little is known yet regarding the recently discovered Insl5. Therefore, future studies are warranted to ascertain whether Insl5 meets the necessary criteria to be considered a regulator of feeding given that the exogenous studies mentioned above utilize high doses of Insl5, although the initial work is promising. For an extensive review of additional neuroendocrine gut peptides regulating food intake, see, ref. ^237^.

## Gut microbiota–brain connection

The gut microbiota consists of over 10 trillion diverse microbes, increasing in density distally through the GI tract^238^. The host and gut microbes exist in a symbiotic relationship, in which the host creates a nutrient-rich environment for microbes, and the microbes, in turn, impact host physiology, immunology, and metabolism^239^. Germ-free (GF) mice, devoid of any microbiome, can provide valuable insight into the impact of the gut microbiome on host metabolism. GF mice have reduced adiposity and increased insulin sensitivity, despite increased food intake^240^, and are protected against diet-induced obesity^241^. GF mice exhibit altered gut–brain metabolic signaling, with changes in EEC number, as well as differences in intestinal nutrient-sensing machinery and nutrient-induced gut peptide release compared to conventional mice^242^. Transcriptomic analysis revealed significant differences in small intestinal GLP-1-secreting L-cells from GF versus conventional mice, including increased GLP-1 content in GF mice, which was rapidly altered following conventionalization, implicating a strong role of the gut microbiota in regulating EEC physiology^243^. In line with this, treatment of cultured cells with several different bacteria decreased GLP-1 mRNA but increased fatty acid sensor GPR120 expression, which induces GLP-1 release^244^.

In addition to impaired EEC signaling and gut peptide release, GF models have impaired ENS activation compared with conventionalized and specific-pathogen-free models^245,246^. In GF mice and in antibiotic-treated mice, which have a very depleted gut microbiota, enteric neuron number and activation are reduced^72,246,247^, likely via impairments in Toll-like receptor 4 (TLR4) signaling. TLR4 is expressed on the ENS, recognizes microbial byproducts such as lipopolysaccharide (LPS), and when activated, initiates a downstream signaling cascade to increase inflammatory cytokine production and immune system activation^247,248^. In addition, ENS maturation and function may be regulated by the production of 5-HT by the gut microbiota^249^. Regardless of the mechanism, these impairments in ENS signaling could lead to alterations in the gut–brain axis that regulate energy homeostasis, as GF mice are GLP-1 resistant, demonstrating reduced activation of brainstem neurons. Importantly, colonization of GF mice with a healthy gut microbiota restores neuronal GLP-1 and ENS signaling pathways in the gut; however, this effect is abolished in mice recolonized with a microbiome from diabetic mice^72^. These studies not only indicate a role for the gut microbiota in impacting gut–brain neuronal signaling, but also suggest that differential gut microbiomes could have varying effects.

**Fig. 3:**
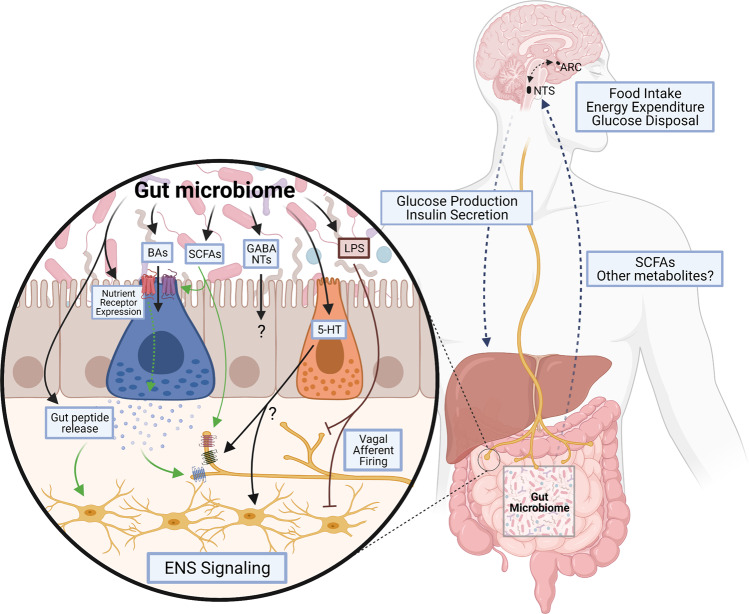
Impact of the microbiome on gut–brain signaling. The microbiota produces several metabolites that impact gut–brain signaling directly and indirectly. The microbiome can directly influence nutrient receptor expression and gut peptide release from EECs and can influence the production of serotonin (5-HT) from ECs, which may alter ENS or vagal afferent activation. Microbially derived or modified metabolites also influence the gut–brain axis. Bile acids (BAs) can alter the expression of TGR5 and regulate gut peptide release from EECs, while SCFAs alter nutrient receptor expression and gut peptide production by EECs or activate vagal afferents directly. Alternatively, metabolites such as LPS, a product of pathogenic microbes, can impair gut–brain signaling by preventing the activation of vagal afferents or the ENS. Overall, the gut microbiome and its metabolites can alter gut–brain signaling to influence the activation of the NTS and ARC, which regulate food intake, energy expenditure, glucose disposal and production, and insulin secretion.

Metabolic diseases are associated with altered microbial functionality, composition, and diversity^250–252^. Transplanting the gut microbiota from either obese or lean mice or humans into GF mice recapitulates the host phenotype, highlighting the potential causality of the gut microbiota in the development of metabolic dysregulation^252,253^. Furthermore, fecal microbiota transplant from lean donors to individuals with metabolic syndrome improves the insulin sensitivity of recipients^254^, demonstrating that altering the gut microbiota could improve metabolic outcomes. Alternatively, beneficially altering the gut microbiota through the use of prebiotics, nondigestible carbohydrates that promote the growth of beneficial bacteria, reduces body weight and adiposity, and improves glucose tolerance in rodents and humans^255–257^. Treatment with inulin or oligofructose increases the production and release of gut peptides, which is associated with reduced food intake and increased satiety^258–261^. Furthermore, the improvements in glucose homeostasis following prebiotic treatment are dependent on GLP-1R signaling, implicating that prebiotic-induced alterations in the microbiome restore the gut–brain axis^257^. In addition to increased secretion of gut peptides, prebiotic treatment improves gut barrier integrity under the conditions of HF feeding and obesity, ultimately reducing circulating LPS^256^. LPS translocation across the epithelium during high-fat feeding leads to metabolic endotoxemia and systemic inflammation^262^. In addition to acting on other metabolically relevant tissues, vagal afferents contain TLR4^263^, and LPS impairs gut–brain vagal signaling^264,265^. LPS reduces leptin-mediated activation of VANs, both in vitro and in vivo^264,265^. Interestingly, vagal CCK signaling is potentiated by vagal leptin signaling^266,267^, and diet-induced leptin resistance in VANs mediates impairments in vagal CCK action^268^. Thus, impaired CCK signaling in HFD-induced obesity may be due to increased LPS-driven leptin resistance at VANs. In line with this, chronic low-dose treatment with LPS increases food intake, decreases CCK-induced satiety, and causes vagal afferent leptin resistance in rats^264^. Therefore, it is possible that the gut microbiota can impact intestinal nutrient-sensing mechanisms (Fig. 3); however, most work to date has focused on the distal gut microbiota, while nutrient-sensing negative feedback takes place in the small intestine.

Recent work has highlighted the small intestinal microbiota as a major mediator of the gut–brain axis in regulating glucose homeostasis. A high-fat diet rapidly shifts the small intestinal gut microbiota, resulting in a drastic reduction in *Lactobacillius*^130^. In parallel with rapid shifts in the small intestinal microbiota, impairments in the regulation of glucose homeostasis via small intestinal lipid sensing occur following just 3 days of HFD feeding^130^. Interestingly, small intestinal microbiota transplant from rats fed either a healthy chow or a HFD restored small intestinal lipid sensing, improving whole-body glucose homeostasis, and vice versa^130^. Similarly, acute metformin treatment restores HFD-induced impairments in small intestinal glucose sensing that regulate hepatic glucose production. This is due to increased small intestinal sodium/glucose cotransporter 1 (SGLT1) expression and subsequent GLP-1 release following intestinal glucose infusion, which are mediated by metformin-induced alterations in the small intestinal microbiota^131^. In line with alterations of the small intestinal microbiota mediating nutrient-induced gut–brain signaling, direct small intestinal infusion of *Lactobacillus gasseri* restores intestinal lipid sensing^49,130^. *Lactobacillus gasseri* expresses bile salt hydrolase, and improvements in lipid sensing were dependent on reduced farnesoid-X receptor (FXR) signaling^130^, highlighting the impact of bile acids on gut–brain signaling mechanisms that regulate metabolic homeostasis.

Bile acids are produced in the liver and excreted into the small intestine to aid in fat digestion^269^. In addition, specific bile acids can act as signaling molecules by activating the transcription factor FXR or binding to G protein-coupled bile acid receptor-1 (TGR5), both of which are expressed in EECs^270^. As such, TGR5 activation stimulates secretion of GLP-1, while FXR activation reduces GLP-1 secretion, demonstrating a complex interplay between the differential bile acid pool composition and gut–brain signaling^270,271^. For example, high-fat feeding alters the small intestinal microbiota to increase intestinal, brain, and circulatory levels of taurine-conjugated bile acids, including and taurochenodeoxycholic acid (TCDCA), which is an FXR agonist^272^. Increased TCDCA leads to insulin resistance that is due to impaired insulin action in the dorsal vagal complex of the hindbrain from increased local TCDCA–FXR signaling^272^. Furthermore, at the intestinal level, TCDCA-mediated activation of FXR leads to impaired nutrient-sensing mechanisms that regulate glucose homeostasis^273^. Interestingly, transplant of the small intestinal microbiome from animals fed regular chow restores both central insulin and intestinal nutrient-sensing signaling mechanisms, highlighting the role of the gut microbiota in shaping bile acid pool composition^272,273^. However, the role of FXR in improving metabolic dysfunction is contentious, as intestine-specific FXR agonism improves the metabolic profiles of mice^274,275^ and increases the expression of TGR5^276^, which mediates bile acid-induced GLP-1 secretion from EECs^277^.

In addition to shaping the bile acid pool, the gut microbiota produces a substantial quantity of metabolites that can have a vast impact on the host. In the gut–brain axis specifically, short-chain fatty acids (SCFAs), butyrate, acetate, and propionate, produced from gut microbiota fermentation of nondigestible fiber, improve both energy and glucose homeostasis in rodent models of obesity and insulin resistance^278,279^. For example, chronic butyrate administration reduces food intake and body weight via a neural gut–brain axis^280^. SCFAs bind to GPR43 and GPR41 and olfactory receptor 558 localized on EECs^281^ and can regulate the number and activity of EECs^282,283^. Treatment of human and mouse small intestine organoid models with SCFAs increases the number of GLP-1-producing cells twofold^284^, and SCFA infusion activates colonic EECs to increase GLP-1 release in mice^281^. SCFA-induced GLP-1 and PYY release is at least partially dependent upon the GPR43 receptor pathway^113,281–283^. However, the exact mechanism of SCFA-induced gut peptide release is controversial, as SCFAs may activate EECs via basolateral sensing mechanisms, similar to LCFAs. For example, while luminal or vascular administration of butyrate during an isolated perfused colon study increased GLP-1 and PYY secretion^93^, only vascular administration of acetate induced GLP-1 and PYY secretion, with luminal acetate inducing only GLP-1 secretion^93^. Interestingly, although vascular SCFAs activate EECs, vascular administration of a GPR43 agonist did not induce gut peptide secretion, and vascular coadministration of butyrate with a GPR41 antagonist did not impede gut peptide secretion^93^. This suggests that GPR43 and GPR41 may not be part of the mechanism required for GLP-1 and PYY release from SCFAs; however, this study did not test luminal GPR43 or GPR41 effects.

In addition, given the close proximity of VANs, SCFAs could directly activate neurons that contain GPR41^285^. Indeed, while SCFAs infused into the intestinal lumen activate VANs projecting from the gut^12^ and intraperitoneal SCFA injection suppressed food intake through vagal afferent signaling, butyrate treatment directly activates isolated nodose ganglion neurons from mice^12^. Thus, given that oral, but not intravenous, administration of butyrate decreased food intake in a manner that is dependent on gut–brain signaling^280^, SCFAs may activate VANs either directly or indirectly via gut peptide release^93,283^. Despite these findings, endogenously produced acetate can also cross the blood–brain barrier and can directly activate energy-regulating neurons to decrease appetite^286^.

The gut microbiota is also a major regulator of 5-HT production. GF mice have decreased 5-HT levels compared to conventional mice, which is rescued following gut microbiota transplantation^287^. Improvements in 5-HT production occur by increasing EC expression of tryptophan hydroxylase, the rate-limiting enzyme in EC 5-HT production^288^, or by increasing microbial metabolites, such as SCFAs and BAs, that are known to stimulate EC 5-HT production^74^. ECs in the gut produce ~95% of the 5-HT in the body, which is presumed to act on 5-HT receptors expressed on local gut neurons^289^. 5-HT administered intravenously^290^ or intraluminally^291^ activates intestinal VANs and myenteric neurons^292,293^ in rats. This 5-HT-mediated vagal afferent activation increases c-Fos expression in the NTS and area postrema^294^, demonstrating the potential gut–brain relay of ECs. More recently, it was shown that ECs act as gut chemosensory cells, similar to other EECs such as GLP-1-secreting cells, and that they form synapse-like connections with primary afferent neurons^295^. Overall, there is extensive evidence indicating that the gut microbiota has a large impact on nutrient-induced gut–brain signaling that regulates metabolic homeostasis (Fig. 3). However, our current understanding likely represents a small proportion of the potential of the gut microbiota to impact metabolic homeostasis via gut–brain signaling, and this is a field that needs to continue to be explored. For example, in addition to 5-HT, bacteria have been found to produce a large number of hormones or neurotransmitters, including dopamine, leptin, and gamma-aminobutyric acid (GABA). Certain *Bifidobacterium* and *Lactobacillus* species can produce GABA^296–300^, and oral treatment with GABA or bacteria that produce GABA improves metabolic parameters in mice^298,301^. However, the impact of bacterially derived GABA, the primary inhibitory brain neurotransmitter, on the CNS is completely unknown.

## Conclusion

Our understanding of how the GI tract contributes to disease, especially the development of obesity and diabetes, is still developing. As detailed in Fig. 1, there is a complex relationship between the gut and the brain. For example, EECs within the GI tract can sense nutrients and release a variety of gut peptides to influence both energy and glucose homeostasis. However, more work is needed to fully understand the mechanisms through which various nutrients activate EECs and release gut peptides (Fig. 2), the metabolic capabilities of specific peptides, and their mechanisms and sites of action. Traditionally, it was thought that gut peptides act on VANs to signal to the brain to mediate their metabolic effects, as detailed above for GLP-1 and CCK. However, recent work has implicated the ENS in mediating the effects of GLP-1, while the role of gut peptide-mediated VAN signaling is controversial. Indeed, with technological and methodological advancements, the intricacy of the gut–brain axis in regulating energy and glucose homeostasis is currently being uncovered. For example, although vagal afferents were once thought to be fairly homogenous in their signaling capacity and function, extensive tracing and single-cell sequencing studies have demonstrated their complexity and diversity^28,31,32^. Although these recent studies appear somewhat conflicting and contentious^28,31,32^, as more studies continue to map out the neuronal gut–brain signaling axis, they could ultimately lead to very precise therapeutics directly targeting specific neural subgroups within vagal afferents to treat specific physiological mechanisms, such as controlling food intake versus intestinal motility. Similarly, given the recent work characterizing the uniqueness and heterogeneity of EECs^81,84–87^, it may also be possible to target specific populations of EECs or even convert certain EECs into cells that secrete specific peptides^302–304^.

Along these lines, our understanding of the impact of the gut microbiota on host gut–brain signaling is still poorly understood, due in part to the current limitations in analyzing the gut microbiome and the plethora of metabolites it produces. In the current review, we detail the ability of the gut microbiome to impact small intestinal nutrient-sensing mechanisms that activate a gut–brain neuronal axis to regulate hepatic glucose production. However, given the potential similarities in gut–brain signaling pathways regulating food intake and hepatic glucose production (detailed above), it is likely that the small intestinal microbiota can also impact food intake and energy homeostasis, although this remains to be assessed. Furthermore, we highlight the strong impact of bacterially derived metabolites, mainly SCFAs and bile acids, on overall metabolic homeostasis (Fig. 3) via activation of EECs or peripheral neurons directly. However, these microbe–host interactions are likely the tip of the iceberg, as the bacteria in the gut contain numerous genes, likely corresponding to an extensive degree of functionality. Therefore, it is very plausible that there are many unrecognized mechanisms through which the gut microbiota could impact gut–brain signaling pathways. In addition to better elucidating the impact of the gut microbiota on host homeostasis, only a small amount has been done to tap into the therapeutic potential of the gut microbiota. For example, with advancements in molecular engineering, scientists are currently attempting to alter the gut microbiota into specific compositions using CRISPR/Cas9 technology. Furthermore, bacteria are being created that contain specific proteins or signaling molecules to achieve site-specific delivery of gut-targeted therapies that can impact metabolic homeostasis^305–307^. These advances could lead to novel therapeutics to target specific microbiota–gut–brain signaling pathways that regulate energy and glucose homeostasis.

Overall, the gut–brain axis holds significant promise as a target for treatment options addressing obesity and diabetes. The gut plays a crucial role in maintaining energy and glucose homeostasis through extensive relays to the brain to inform the energy-regulating centers about incoming nutrient compositions. Although this review focused on the ability of gut-derived signals to control food intake, there is evidence that the gut and gut microbiota can also impact energy expenditure, although this requires more investigation^308–310^. Importantly, obesity and diabetes are associated with impairments in gut–brain signaling^68,311^, and the success of both surgical and pharmacological treatments for both diseases is due at least in part to alterations in the gut–brain axis^6,7,10,131^. With advances in our understanding of how these specific gut-derived relay signals contribute to food intake, energy expenditure, and glucose homeostasis, the potential to target these pathways in the treatment of metabolic disease will grow.
